# Inter-individual variability and modeling of electrical activity: a possible new approach to explore cardiac safety?

**DOI:** 10.1038/srep37948

**Published:** 2016-11-30

**Authors:** Jean-Yves Le Guennec, Jérôme Thireau, Aude Ouillé, Julien Roussel, Jérôme Roy, Serge Richard, Sylvain Richard, Eric Martel, Pascal Champéroux

**Affiliations:** 1 Inserm U1046-UMR CNRS 9214, Université de Montpellier, 371 avenue du doyen Gaston Giraud, 34295 Montpellier (France); 2 Centre de Recherche Biologique, CERB, Chemin de Montifault, 18800 Baugy (France)

## Abstract

Safety pharmacology aims to predict rare side effects of new drugs. We explored whether rare pro-arrhythmic effects could be linked to the variability of the effects of these drugs on ion currents and whether taking into consideration this variability in computational models could help to better detect and predict cardiac side effects. For this purpose, we evaluated how intra- and inter-individual variability influences the effect of hERG inhibition on both the action potential duration and the occurrence of arrhythmias. Using two computer simulation models of human action potentials (endocardial and Purkinje cells), we analyzed the contribution of two biological parameters on the pro-arrhythmic effects of several hERG channel blockers: (i) spermine concentration, which varies with metabolic status, and (ii) L-type calcium conductance, which varies due to single nucleotide polymorphisms or mutations. By varying these parameters, we were able to induce arrhythmias in 1 out of 16 simulations although conventional modeling methods to detect pro-arrhythmic molecules failed. On the basis of our results, taking into consideration only 2 parameters subjected to intra- and inter-individual variability, we propose that *in silico* computer modeling may help to better define the risks of new drug candidates at early stages of pre-clinical development.

Cellular action potentials (APs) drive rhythmic heart contraction. APs depend on ion channel activity, and repolarization, which triggers life-threatening arrhythmia, is of major concern. In humans, AP duration (APD) depends essentially on ionic currents such as I_CaL_ for the plateau phase and on I_K1_, I_KR_ (the hERG channel) and I_KS_ for the repolarization phase and the resting membrane potential^1^. The QT interval as measured on an electrocardiogram reflects the depolarization of the ventricles and is thus an indicator of the ventricular APD.

Many drugs, both cardiovascular and non-cardiovascular, are cardiac ion channel blockers. This mode of action, expected or undesirable, often modulates the initiation or electrical propagation of the AP and can initiate or promote life-threatening ventricular arrhythmias (VAs). In safety pharmacology, the most widely studied drug-induced cardiac side effect is Torsade de pointe (TdP), a rare but lethal tachyarrhythmia^2^. The mechanisms underlying the occurrence of such VAs are complex and not fully understood, but TdP is often associated with long QT syndrome (LQTS), either inherited or acquired, involving the dysfunction of ion channels or associated regulatory proteins.

The current hypothesis is that the prolongation of the ventricular APD provides the conditions for early after-depolarizations (EADs), which trigger TdP preceding ventricular fibrillation^3^. Thus, all conditions that favor AP prolongation (or QT prolongation) are breeding grounds for the triggering of TdP^4^,^5^. Fortunately, TdP is rarely induced in the general population even by classes of molecules known to induce QT interval lengthening. Different factors may explain this low frequency of occurrence. One possible explanation is metabolic status, which participates in both intra- and inter-individual variability. For example, in diabetes in humans, it is hypothesized that “dead-in-bed syndrome” is linked to the lengthening of the QTc interval during hypoglycemia^6^,^7^. Similarly, hyperglycemia potentiates dofetilide-induced APD prolongation in the guinea pig heart, possibly reflecting the limited capacity of P-glycoprotein to eliminate/eject the drug^8^. Thus, metabolic state indirectly modulates the APD and can impact the effects of drugs. Among the diversity of metabolic parameters, glycemia is not the only one that can interfere with drug effects on cardiac electrical activity. Polyamines, mainly spermine (SPM), are known to directly regulate the inward-rectifier current I_K1_ and thus impede rapid repolarization during the final phase of the cardiac action potential (AP)^9^. Decreased I_K1_ is known to be involved in the genesis of ventricular arrhythmias^10^. Blood SPM concentrations vary with diet^11^. This variation is amplified in elderly people whose ability to metabolize SPM is lower, and could thus amplify the consequences of a drug that inhibits the conductance G_KR_ to the APD^12^.

In safety pharmacology, while a large number of models have been developed to detect rare torsadogenic molecules, torsadogenicity by itself is not tested in most cases. Instead, surrogate markers suspected of being linked to arrhythmias such as QT prolongation are studied^2^. In this way, Kui *et al*. recently developed an *in vitro* model for cardiac safety screening using isolated guinea pig hearts and looking at QTc prolongation by drugs inhibiting I_KS_^13^. A new proposal is currently being formulated to evaluate the utility of computational tools for regulatory decision making (Comprehensive *In Vitro* Proarrhythmia Assay, CiPA). However it relies on the same principle, i.e. the detection of surrogate markers of TdP, using three distinct sequential assays of a panel of heterologously expressed human ventricular ion channels on a computationally generated (in silico) human ventricular AP and on a human stem-cell-derived cardiomyocyte model, to identify electrophysiological effects^14^,^15^. Recently, Davies *et al*. have reviewed mathematical tools that can be used for the cardiovascular safety evaluation of compounds and decision making^16^. In the final analysis, drugs that induce QT prolongation, and possibly TdP, are most frequently identified as potent inhibitors of the hERG channel (I_KR_). This is why the hERG assay is a regulatory test in drug development^17^. However, all drugs blocking the hERG channel are not systematically associated with TdP, suggesting that I_KR_ blockade and QT prolongation are insufficient to trigger TdP^18^. However, the fact that some I_KR_ inhibitors are torsadogenic has slowed the development of promising new drugs^19^,^20^.

It is thus important to develop new tools to discriminate potentially lethal compounds from safe drugs. One such tool is *in silico* computer modeling based on multichannel assays^21^,^22^,^23^. This method reduces the number of animals used for preclinical studies. There are still some problems with using such models to predict the potential arrhythmogenic properties of new chemical entities (NCE). For example, Romero *et al*.^24^ used the ventricular cardiac cell model developed by Ten Tusscher and Panvilov^25^ to evaluate its relevance in testing changes in the conductance and kinetics of the main ion currents involved in ventricular repolarization. They also extended their investigations to modifications of preclinical biomarkers of arrhythmia risk such as APD, triangulation of the AP and the APD restitution curve. While their model could reproduce most experimental findings, some discrepancies, mainly quantitative, remained suggesting that the model needed to be improved with new experimental input. Nevertheless, such models are sufficiently advanced to provide helpful information in safety pharmacology^26^. Mirams *et al*. studied the effects of 31 known drugs on different ventricular cell models based on the reported effects of these molecules on I_Na_, I_CaL_ and I_hERG_^23^. This approach was relatively successful although there was still a false negative in the study. This is important since the main objective of safety pharmacology is to identify the potential of an NCE to induce a rare lethal event as early as possible. Another weakness of this study, which can be easily solved, is that other currents known to be potentially involved in drug-induced arrhythmias, such as I_KS_ or I_K1,_ were not included in the tests^21^,^27^.

An important limitation of computer models could be the failure to observe drug-induced arrhythmias. In all modeling studies^23^,^24^,^28^, the end-points are still surrogate markers such as AP prolongation, triangulation of the AP, etc. The difficulty of models to reproduce drug-induced arrhythmias, besides their accuracy^24^, is that they do not reproduce the inter-individual variability caused by different physiological and pathological statuses. When a ventricular cell model is run with fixed control parameters, the shape and duration of the AP are always the same whereas, strikingly, the QT interval measured in a sample of the general population (n = 76) varies widely, between 320 and 460 ms^29^. This problem has begun to be examined by several groups. Walmsley *et al*. have already underlined the occurrence of variability in the APD and proposed a mathematical solution by adding some physiological “noise” to the parameters used to simulate AP variations^30^. Even though it is interesting, this approach is not of much use to pharmacology in general and safety pharmacology in particular because the variability (“noise”) added has no physiological relevance. At the same time, Sobie was the first to use multivariable regression to introduce variability to the electrophysiological parameters (electrophysiological ion channel properties) of a cellular ventricular AP model^31^. His method has since been used to evaluate the APD before and after hERG inhibition^27^,^32^. He was able to induce variable AP prolongation whatever the initial APD, observed experimentally or clinically. Other authors focused on the influence of the passive properties of the cardiac cell membrane on safety assessment, but here again, the approach was mainly statistical and did not rely on experimental data^33^,^34^. More recently, Britton *et al*. have proposed the use of calibrated models^35^. These models are obtained by randomly assigning parameter values (ion conductance and kinetics) and comparing the AP simulation results with experimental recordings. Of 10,000 models tested, 213 were considered relevant. However, this interesting approach is still mainly mathematical. Indeed, even if there is an acceptable comparison with experimentally observed AP, there is no evidence that the parameters used *in silico* fit with the experimental parameters and inter-individual variability of the general population (see ref. 36).

Intra- and inter-individual variability in the response to drugs are well-known^37^. In the case of cardiovascular drugs, this variability is assumed to be mainly of pharmacokinetic or pharmacodynamic origin and even to be linked to sex differences^37^,^38^,^39^. Whatever the cause (genetics, sex, ageing, diet, drug-drug interactions…), the consequence is a change in the circulating concentrations of the drug or its active metabolites^37^,^38^. A way to bypass this potential problem is to study the electrophysiological effects of an NCE at the highest soluble concentration^40^. However, even in this case, there is still some inter-individual variability that is not due to variations in circulating drug concentrations but to other factors such as single nucleotide polymorphisms (SNPs), mutations or environmental/metabolic factors (physiological or pathophysiological). For example, mutations or SNPs in the L-type calcium channel, leading to variable calcium conductance, have already been associated with alterations of ventricular repolarization, LQTS and arrhythmia^41^. Also, it has been shown recently that impaired inactivation of the L-Type Ca^2+^ current is a potential mechanism for the variable arrhythmogenic risk of hERG-channel-blocking drugs^42^.

In their review, Corrias *et al*. have proposed the use of computer simulations for cardiac safety experiments^28^ as has Denis Noble^26^. In Corrias’s paper, the authors introduce the concept of channel property variability in computer simulations, not for the reproduction of inter-individual variability for a given species but rather to explain the differences between species in developing drug-induced arrhythmias. Nevertheless, the criteria for pro-arrhythmic risk are still arrhythmic biomarkers such as APD and QT prolongation.

In the present study, we evaluated how variability of L-type calcium channel conductance and of a single metabolic parameter (spermine concentration) influences APD and the induction of arrhythmias by 11 representative QT-prolonging compounds, using two different *in silico* models of the human AP (endocardial and Purkinje cells). The pharmacological properties of these hERG-blockers, as described in the literature^21^,^43^ are summarized in Fig. 1.

## Results

**Figure 1:**
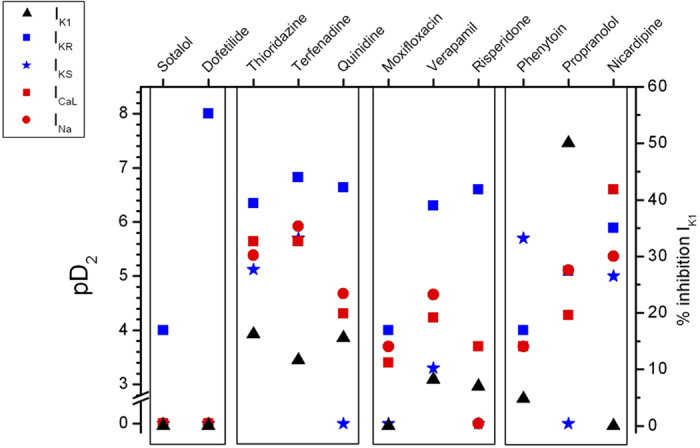
Effects of molecules from subgroup A1 (dofetilide and sotalol), subgroup A2 (terfenadine, thioridazine and quinidine), group B (moxifloxacin, verapamil, risperidone), and group C (Phenytoin, propranolol, nicardipine) on I_KR_, I_KS_, I_K1_, I_Na_ and I_CaL_ currents (taken from ref. 14). The inhibition of I_K1_ is expressed in % on the right scale while for the other currents, the IC_50_ (as given by pD2 = (−Log(IC_50_)) is represented on the left scale.

**Figure 2:**
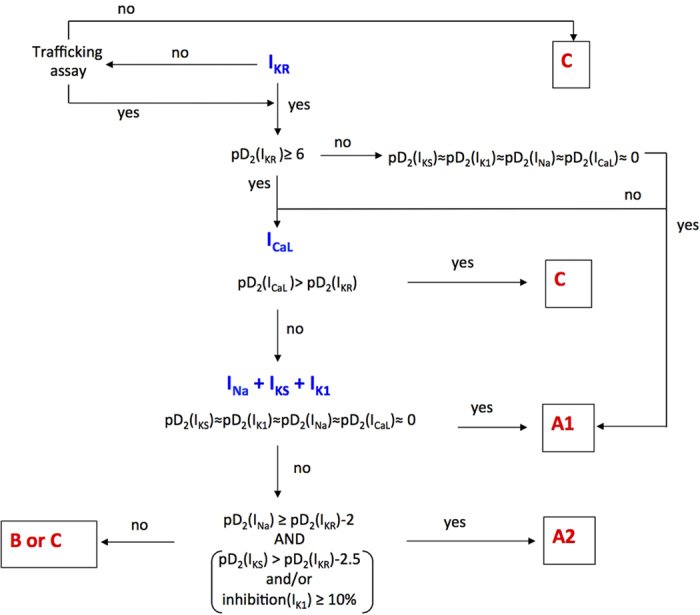
Decision tree proposed to classify molecules using TdPScreen ranking^26^.

**Figure 3:**
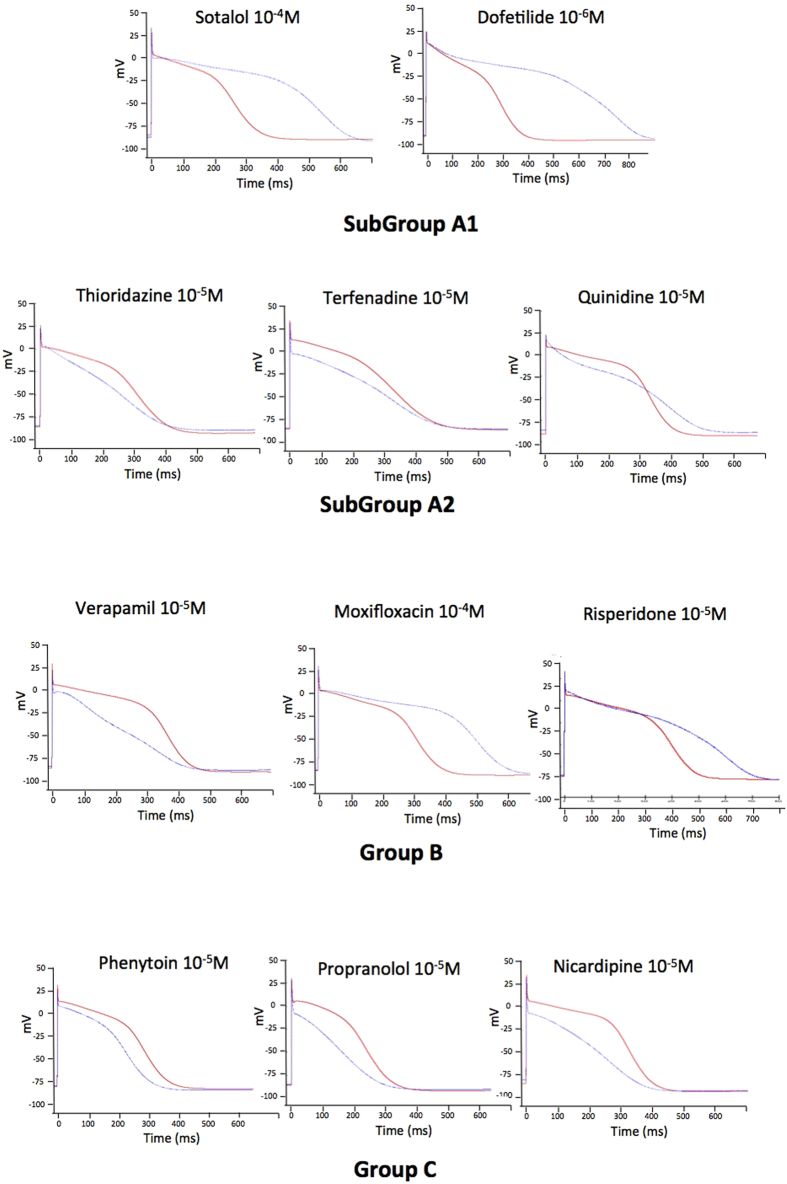
Typical effects of drugs on the AP recorded from isolated dog Purkinje fibers. APs were triggered by field stimulation at 0.33 Hz in Tyrode (red line) and during drug application at the concentration indicated (black line). Drugs of subgroup A1 cause an exacerbated (>40%) increase in AP duration. Molecules of subgroup A2 cause a triangulation of the delayed phase of repolarization without shortening AP. Compounds of group B either induce (moxifloxacin) or don’t induce AP prolongation (verapamil) and are associated with arrhythmias at a very low frequency (≤2 cases). Group C molecules do not trigger AP prolongation.

**Figure 4:**
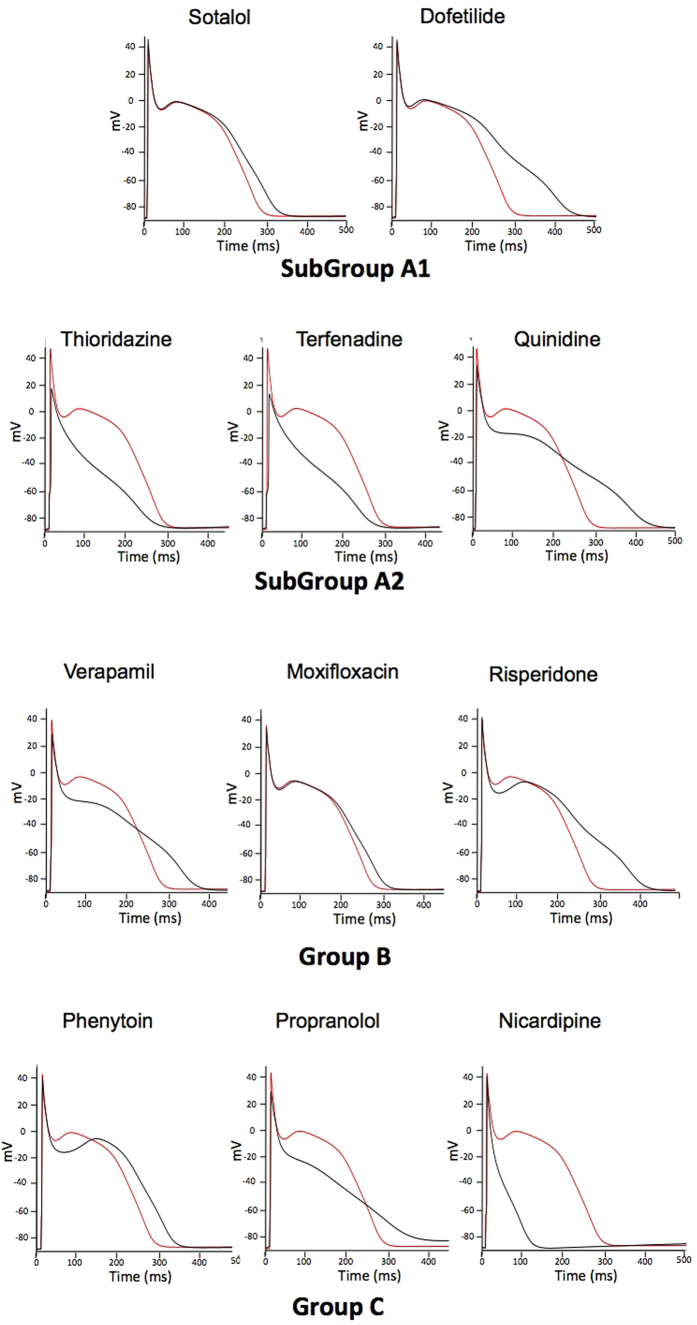
Simulations of human ventricular action potentials under control conditions (red line) and in the presence of the drugs indicated above (black line). To calculate the conductances of I_CaL_, I_Na_, I_K1_, I_KS_ and I_KR_, the conductance block equation (1) given in the Methods section was applied, using the concentrations and IC_50_ (or % of I_K1_ blockade) given in Fig. 1.

**Figure 5:**
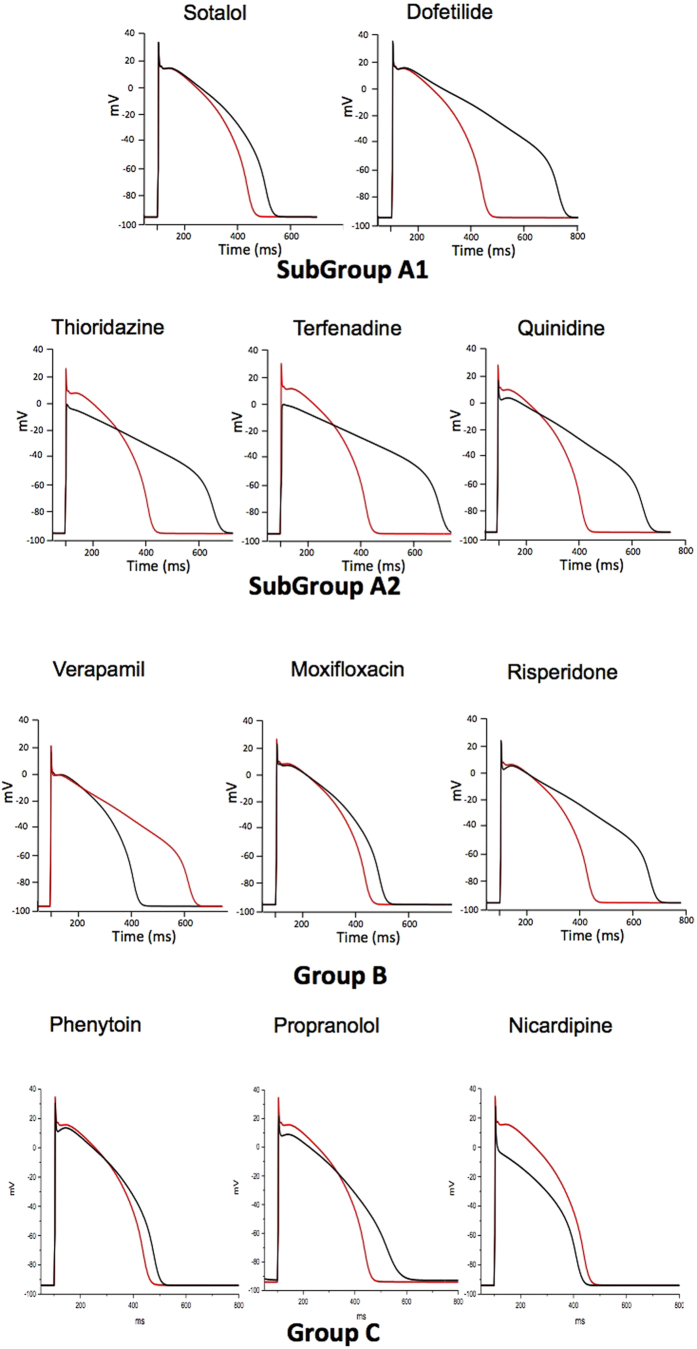
Simulations of ventricular action potentials in the presence of intracellular spermine (SPM) as shown above the graphs and using the G_CaL_ given at left. Simulations were performed under control conditions (red line) and after 90% blockade of G_KR_.

In order to differentiate between deleterious and safe molecules, a standard approach consists of classifying molecules according to a decision tree. Indeed, based solely on the inhibition profile of different compounds acting on different ion channels (Fig. 1), it is difficult to uncover a typical profile that would enable a safe molecule to be distinguished from a potentially pro-arrhythmic one. For example, if we take sotalol and propranolol, two beta-blockers, we can observe that propranolol has stronger I_KR_ blocking properties than sotalol although it is safer. We therefore attempted to build a decision tree (Fig. 2) by refining an algorithm based on AP experiments (TdPscreen^®^) using patch-clamp data obtained with 11 drugs^21^,^40^. The first assay attempted to evaluate the effect of the molecule on I_KR_ and to deduce the half-maximal inhibitory concentration (IC_50_), a measure of the effectiveness of a substance in inhibiting the current. If negative (i.e. if the molecule does not block the hERG current), the probability that the molecule is safe could be considered as very high although a possible trafficking effect (such as for pentamidin, which modifies current density without modifying ion conductance) cannot be excluded. In contrast, if the pD2 (negative log of IC_50_ = −Log(IC_50_)) were lower than 6, all the other currents (I_KS_, I_K1_, I_NA_ and I_CaL_) would have to be tested. If none of these currents were blocked by the compound, the molecule could be considered a pure I_KR_ blocker and thus potentially belong to the A1 subgroup (i.e. an exacerbator (>40%) of the APD). If the pD2 for I_KR_ were 6 or higher, the compound would have to be tested on I_CaL_ only. A pD2 for I_CaL_ higher than that for I_KR_ indicates a drug belonging to group C (i.e. one that would not trigger AP prolongation), meaning that the decrease in the repolarizing current was compensated for by a decrease in the depolarizing current. If not, the effects on the other currents, I_Na_, I_KS_ and I_K1_ would have to be evaluated. If currents other than I_KR_ and I_CaL_ were sensitive, a more complex test would have to be performed to discriminate between A2 and B or C molecules. However, an analysis based on such an algorithm has a major weakness: it relies on the analysis of only 11 molecules, although the probability that other cases not considered in the decision tree exist is not null. Furthermore, it is quite difficult to understand the molecular mechanisms supporting this algorithm. Thus, to understand the possible interactions between a differential blockade of an ion channel and the AP, it would be of use to do some modeling. To compare the results of the modeling with experimental results, dog Purkinje action potentials in control and in presence of the different drugs are shown on Fig. 3 (0.3 Hz stimulating rate). Using the conductance-block formula (see Methods) and the IC_50_ of the drugs for the different channels^21^, we performed simulations using both a human Purkinje cell^44^ (Fig. 4) and a human ventricular cell^45^ model (Fig. 5), at a low stimulation frequency (0.5 Hz) to prolong the APD and avoid false negative results. The simulations were able to reproduce the experimental results obtained with dog Purkinje APs under control conditions and in the presence of the different drugs. This is particularly true for A2 molecules (i.e. molecules that induce a triangulation of the repolarization phase without shortening of the AP). However, major differences appeared. First, blocking only half (sotalol) or more (dofetilide) of the G_KR_ induced only a moderate prolongation of the AP. Secondly, the results of some simulations differed substantially from experimental results, such as in the case of the effect of verapamil on the Purkinje cell model or of propranolol and phenytoin on both models. Finally, a major disadvantage of the models was that we know that some drugs, such as thioridazine or terfenadine, can induce arrhythmias and sudden death in the clinic^46^ that cannot be experimentally reproduced. There are two major reasons for this: (1) model parameters are not precise enough and are set near the mean of a given population. Thus, populations at risk (in other words, individuals who are subject to TdP induction) cannot be excluded from the prediction; (2) the arrhythmias have a multicellular origin, like reentry, in which a cardiac structural component is commonly involved.

**Figure 6:**
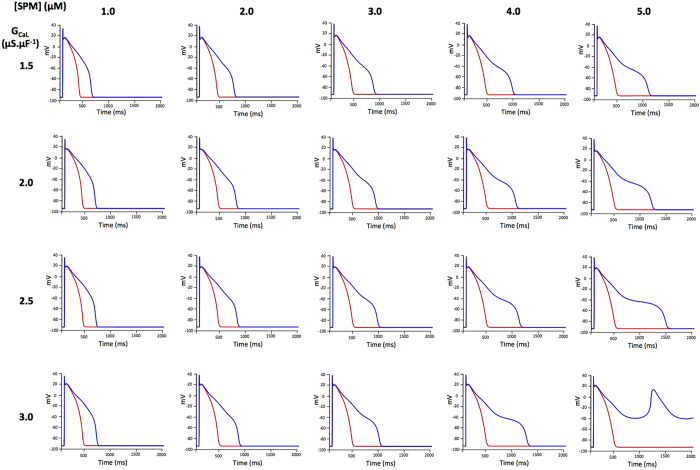
Simulations of human Purkinje cell action potentials in control (red line) and in the putative presence of the drugs indicated above (black line). To calculate the conductances of I_CaL_, I_Na_, I_K1_, I_KS_ and I_KR_, the conductance block equation described in the Methods section was applied, using the concentrations and IC_50_ (or % of I_K1_ blockade) given in Fig. 1.

**Figure 7:**
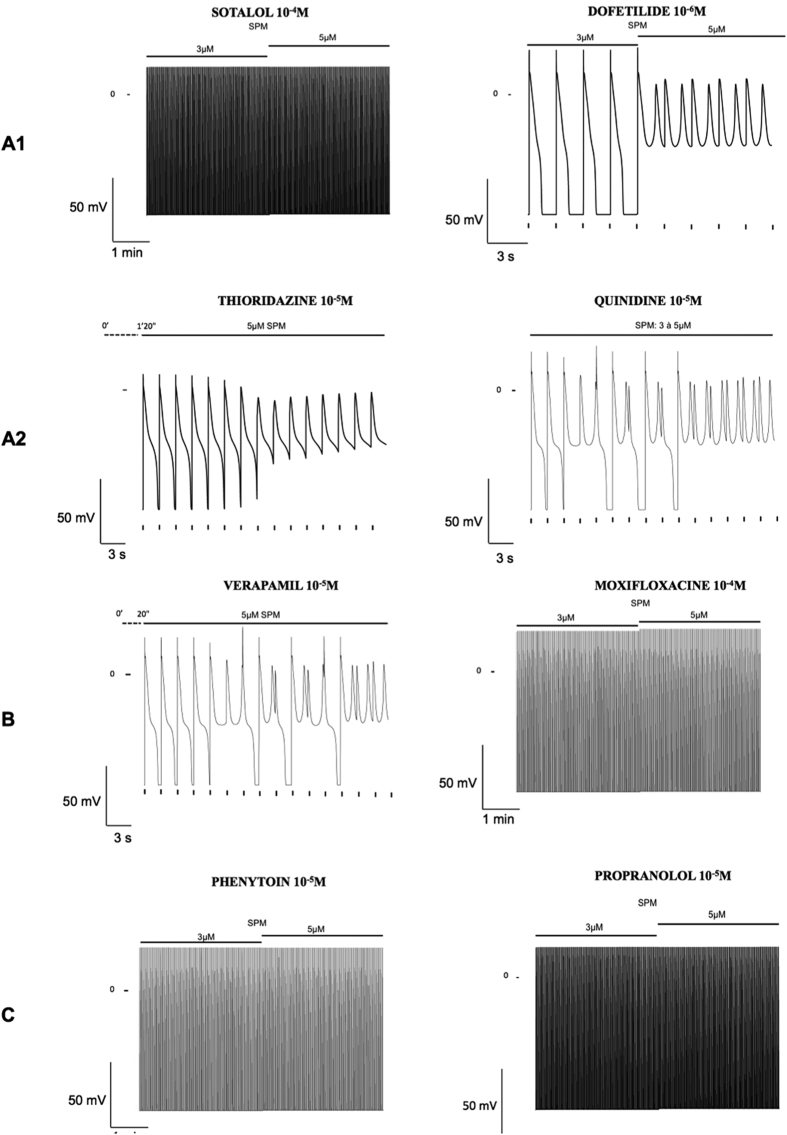
Simulations of the effects of drugs from different TdPScreen^®^ groups (as indicated on the left) after increasing the intracellular concentration of SPM from 3 to 5 μM. Cells were stimulated at 0.5 Hz for 3 min at each SPM concentration. For sotalol, moxifloxacine, phenytoin and propranolol, no arrhythmia occurred during the 6 min-simulation, whatever the [SPM]. For quinidine, an arrhythmia occurred during the transition from 3 μM to 5 μM [SPM]. For thioridazine and verapamil, the transition to 3 μM to 5 μM [SPM] took place 80 s and 20 s respectively before the model triggered arrhythmia. AP was not shown under 3 μM [SPM].

We thus tried not to exclude these parts of the population from the simulation by taking into account some variability between individuals. SPM regulates the outward current I_K1_^47^ and its circulating concentrations vary with age and with diet^11^. SPM concentrations can vary 5 fold^11^. Thus, we varied its concentration in the same range around the mean concentration (3 μM). We also varied G_CaL_ by a factor of two, which is also typically observed^48^ (Fig. 6). For each condition, we observed a control AP that looked normal, but the effects of 90% I_KR_ inhibition differed. In one case out of 16 simulations, we observed the occurrence of Early After Depolarizations (EAD) (Fig. 6). Arrhythmias in patients can appear some time after the treatment has started. Many hypotheses have been put forward for this, such as the accumulation of the drug in tissues, the involvement of metabolites and effects on the transcription/translation of ion channel proteins^43^. Another possibility could be changes in metabolism. Here again, we simulated the effects of different drugs on human ventricular cell models paced at 0.5 Hz for 6 min. During the initial 3 min, the intracellular concentration of SPM was set to the mean value of 3 μM. Then, this concentration was increased less than two fold to 5 μM. As shown in Fig. 7, this change was associated with the triggering of spontaneous arrhythmias by known pro-arrhythmic drugs such as dofetilide, thioridazine, quinidine and verapamil, while it was devoid of effect with safer drugs such as moxifloxacin, phenytoin and propranolol. In addition, the effect of sotalol was not sensitive to the change in SPM concentration.

## Discussion

In the present paper, we tested whether integrating inter-individual variability of different origins (metabolism, conductance) into mathematical models could be of particular interest in order to better predict electrocardiographic side effects and to assess the population-based risk of arrhythmia of NCEs in preclinical development. Our working hypotheses to translate such variability into a predictive mathematical model are based mainly on the presence of SNPs that induce different enzymatic activities^37^,^49^,^50^, leading to variable concentrations of the active compounds or metabolites. A comparable approach has been used successfully to describe inter-individual variability in pharmacokinetics using virtual human populations^37^.

**Figure 8:**
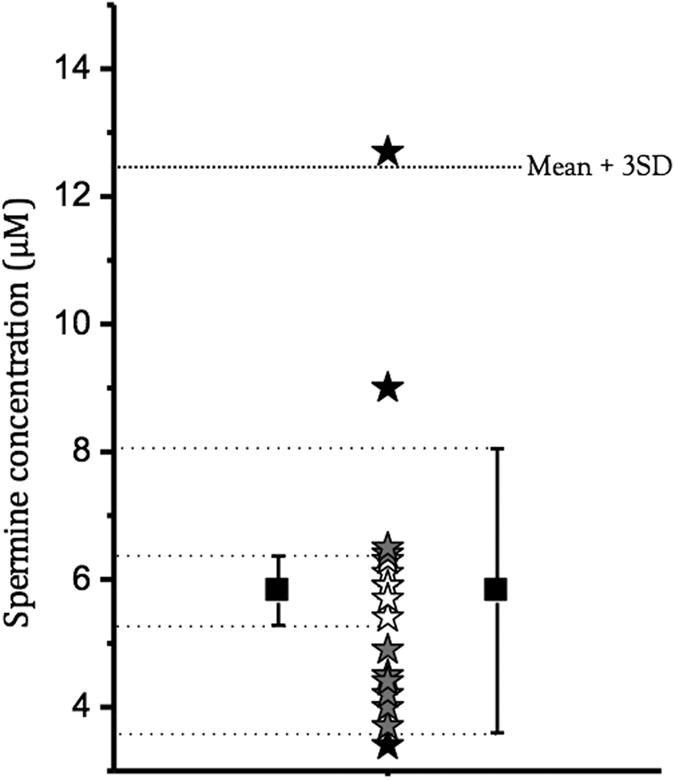
The distribution of circulating spermine concentrations in different people before and after a two-month polyamine-rich soybean diet (taken from ref. 11) are plotted on the same graph to assess the variability of this parameter. Stars in white are in the mean ± SD range (as shown on the right), stars in grey are in the mean ± SEM range (as shown on the left), stars in black are outside the previous ranges. The upper limit of mean + 3SD is indicated, and corresponds to a probability of measuring a similar or lower SPM concentration in 99.8% of the general population.

By varying calcium conductance from 1.5 to 3 pS/pF, we indirectly assessed the consequences of the presence of SNPs in calcium channels. This can be done for all the other channels involved in the AP using a multivariable regression approach^32^ based on experimental data^51^, coupled or not to a mathematical determination of the distribution in the population to increase the representativity of the model. For hERG channels, we assessed the effects of different SNPs on initial electrophysiological properties and drug sensitivity, but the 9 SNPs evaluated did not show strong differences with the 48 drugs tested^34^. Another source of variability could be metabolic variations, as already shown for glycemia. Another example of the numerous parameters that could vary with metabolism is polyamine concentration^32^. In a study involving 17 subjects, Soda *et al*. showed that the circulating concentration of polyamines varies with diet, with an almost five-fold difference between the lowest and the highest level^11^. Also, if we use a single graph to plot individual values and the mean and Standard Error of the Mean (SEM) and Standard Deviation (SD) (Fig. 8), we can see that the highest values are quite far above the mean, by more than +3SD in one case. In our computer simulations, this value could represent the singular point that could lead to rare arrhythmias. The regulation of ion channel activity by polyamines is well known and is implicated in differences in the shape of I_K1_ in the atrium and the ventricle, leading to specific AP waveforms in both tissues^52^. Indeed, there is a plateau in the ventricular AP (responsible for the QT interval) due to the negative slope of the outward I_K1_-V curve. The rectification of I_K1_ takes place due to SPM and Mg^2+^, and the concentration of SPM is different in the atrium and ventricle, which could explain the difference in rectification^47^. Sarkar and Sobie have suggested that G_K1_ is of the same importance as G_KR_ in the repolarization reserve^12^. Thus, in an individual with a lower G_K1_, a drug-induced inhibition of G_KR_ could be deleterious. The case of polyamines and the inward rectification of I_K1_ is of interest in the context of the present work in many respects. The levels of polyamines depend on diet. They may also vary under various pathological conditions^53^. They increase up to 3-fold during hypertrophy induced by pressure or volume stress, hypoxia, thyroxine or adrenergic agonists^54^. Also, Mg^2+^, which participates in the regulation of inward rectification^41^, can vary in different disease states such as ischemia, heart failure and cardiac hypertrophy, and participates in the triggering of arrhythmias^10^,^55^.

**Figure 9:**
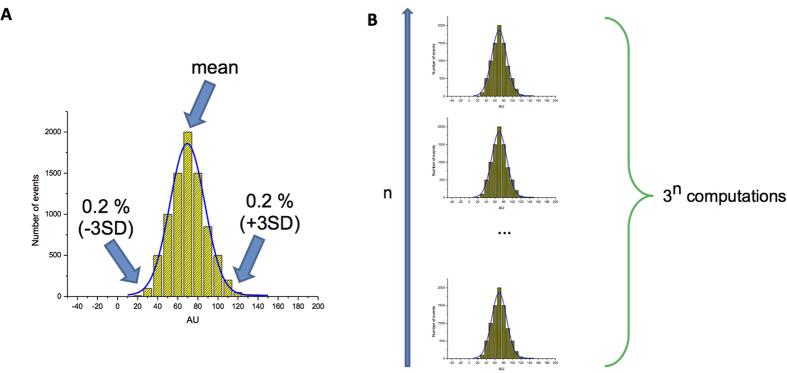
Putative Gaussian distribution of electrophysiological and metabolic parameters. (**A**) idealized distribution of a parameter influencing AP parameters and the position of the three values to be used in simulations. (**B**) proposed simulations using the three values from n parameters.

If we could determine the distribution of both the different electrophysiological parameters and the metabolic variables known to modulate AP properties, we could imagine computations using mean and extreme values representing 0.2% (above or under mean ± 3SD) of the population for each parameter, to predict the effects of an NCE on the main ion currents. With n parameters under study, this would give 3^n^ computations, which is feasible given the current power of computers. The probability of observing arrhythmias in the population under study could be calculated as being 0.002^n’^ where n’ represents the number of parameters corresponding to the 0.2% of the population having the extreme distribution (Fig. 9).

With respect to study limitations (the low stimulation rate known to enhance pro-arrhythmic effects and the use of only two simulation models among several proposed in the literature), the next step would be to do the same in more integrated models, because sodium-channel-blocking properties are a characteristic of the majority of pro-arrhythmic drugs, in addition to I_KR_ inhibition^21^,^56^. Since the main role of this current, besides excitability, is on conduction, only multicellular models could provide insights as to the effect of the inhibition of this current. Our present results also suggest that the combination of experimental data and validated mathematical models could offer a new approach for decision-making in drug discovery and development. Thus, based on our results and in accord with the development of personalized medicine, we propose a new approach to evaluate the propensity of NCEs to induce ventricular arrhythmias in the general population and in subpopulations.

The systematic rejection of drugs that block I_KR_ has led and will always lead to the abandonment of potentially beneficial compounds^57^,^58^,^59^. It is thus important to better apprehend the origins of drug-induced arrhythmias rather than to systematically reject NCE that block I_KR_ with apparent low IC_50_. Thus, new models must be developed to take into account the multiple sources of variability. Our method can be considered as the first step towards the inclusion of a more complete set of parameters that reflect the metabolic status and the variability of ion conductance in order to use properly mathematical models for the detection of the pro-arrhythmic potential of NCEs.

## Methods

### Clinical risk classification

In 2005, we published an algorithm based on a predictive database, TdPScreen™, which compared reference compounds tested in an *in vitro* model utilizing canine Purkinje fibers with clinical data available on the same compounds^40^,^43^. This algorithm enabled a classification of drugs, according to their electrophysiological effects, into three groups. Group A compounds that either exaggeratedly prolong the AP (subgroup A1) or induce a triangulation of the AP without APD shortening (subgroup A2). The reference compounds in this group are the most torsadogenic molecules. Group B compounds induce unexacerbated APD prolongation or a triangulation with APD shortening. The reference compounds in this group are drugs causing QT prolongation without TdP or TdP at a very low frequency (≤2 cases). Group C compounds induce APD shortening or have no effect at all.

### Compounds studied

To investigate multichannel effects, we performed simulations of the effects of 11 compounds, knowing the IC_50_ for inhibition of I_CaL_, I_Na_, I_K1_, I_KS_ and I_KR_^21^. The molecules are quinidine (class I antiarrhythmic), propranolol (class II antiarrhythmic), sotalol and dofetilide (class III antiarrhythmics with β-blocker properties as for class II), verapamil and nicardipine (antihypertensive drugs, calcium blockers), thioridazine and risperidone (antipsychotics), terfenadine (antihistamine, withdrawn from the market), moxifloxacin (antibiotic) and phenytoin (anticonvulsant).

### Computer modeling

The models used in this study are the endocardial cell model described by Fink *et al*.^45^ and the Purkinje cell model developed by Stewart *et al*.^44^. These models are available online from the CellML repository (http://www.cellML.org) and were developed with Cellular Open Resource^60^. To incorporate the effects of channel blockade by molecules, we used the “conductance-block” formula^23^:


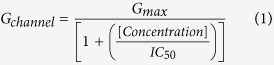


The conductance of the channel in the presence of the molecule, G_channel_, is the drug-free maximal conductance, G_max_, reduced by a factor which is a function of the IC_50_ value of the drug for this channel and the concentration of the drug ([Concentration]). For all compounds and channels in this study, we assumed a Hill coefficient of n = 1^23^.

The stimulation frequencies were chosen to be quite low (0.3 Hz for Purkinje cell modeling and 0.5 Hz for human endocardial cell modeling) since it is known that there is a propensity for drugs that prolong the APD to induce EAD and arrhythmic events initiating TdP and ventricular fibrillation when the heart rate is low, a mechanism also named bradycardia-dependent Torsades de pointes.

## Additional Information

**How to cite this article**: Le Guennec J.-Y. *et al*. Inter-individual variability and modelling of electrical activity: a possible new approach to explore cardiac safety? *Sci. Rep.* **6**, 37948; doi: 10.1038/srep37948 (2016).

**Publisher's note:** Springer Nature remains neutral with regard to jurisdictional claims in published maps and institutional affiliations.
