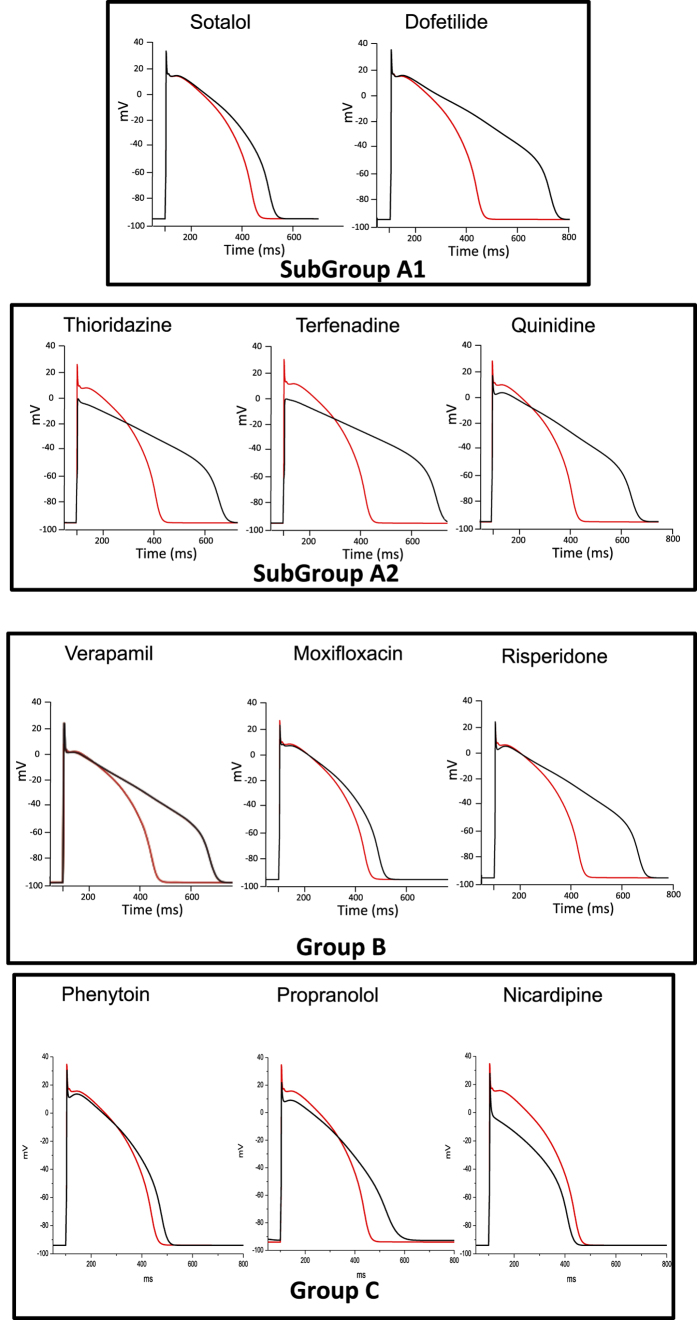# Corrigendum: Inter-individual variability and modeling of electrical activity: a possible new approach to explore cardiac safety?

**DOI:** 10.1038/srep42828

**Published:** 2017-02-16

**Authors:** Jean-Yves Le Guennec, Jérôme Thireau, Aude Ouillé, Julien Roussel, Jérôme Roy, Serge Richard, Sylvain Richard, Eric Martel, Pascal Champéroux

Scientific Reports 6: Article number: 3794810.1038/srep37948; published online: 11 30 2016; updated: 02 16 2017

This Article contains an error in the plot for Verapamil in Figure 5, where the red and black lines representing simulations of human ventricular action potentials under control conditions and in the presence of the drugs have been inverted. The correct Figure 5 appears below as Figure 1.

In addition, there are errors in the order of the legends of Figures 4, 5 and 6. The correct legends are:

Figure 4

Simulations of human Purkinje cell action potentials in control (red line) and in the putative presence of the drugs indicated above (black line). To calculate the conductances of I_CaL_, I_Na_, I_K1_, I_KS_ and I_KR_, the conductance block equation described in the Methods section was applied, using the concentrations and IC_50_ (or % of I_K1_ blockade) given in Fig. 1.

Figure 5

Simulations of human ventricular action potentials under control conditions (red line) and in the presence of the drugs indicated above (black line). To calculate the conductances of I_CaL_, I_Na_, I_K1_, I_KS_ and I_KR_, the conductance block equation (1) given in the Methods section was applied, using the concentrations and IC_50_ (or % of I_K1_ blockade) given in Fig. 1.

Figure 6

Simulations of ventricular action potentials in the presence of intracellular spermine (SPM) as shown above the graphs and using the G_CaL_ given at left. Simulations were performed under control conditions (red line) and after 90% blockade of G_KR_.

## Figures and Tables

**Figure 1:**